# Systematic truncations of chromosome 4 and their responses to antifungals in *Candida albicans*

**DOI:** 10.1186/s43141-021-00197-0

**Published:** 2021-06-21

**Authors:** Wasim Uddin, Darshan Dhabalia, S. M. Udaya Prakash, M. Anaul Kabir

**Affiliations:** Molecular Genetics Laboratory, School of Biotechnology, National Institute of Technology Calicut, Calicut, Kerala 673601 India

**Keywords:** *Candida albicans*, Antifungals, Minimum inhibitory concentration, Chromosome 4, Drug tolerance

## Abstract

**Background:**

*Candida albicans* is an opportunistic human fungal pathogen responsible for superficial and systemic life-threatening infections. Treating these infections is challenging as many clinical isolates show increased drug resistance to antifungals. Chromosome (Chr) 4 monosomy was implicated in a fluconazole-resistant mutant. However, exposure to fluconazole adversely affects *Candida* cells and can generate numerous mutations. Hence, the present study aimed to truncate Chr4 and challenge the generated *Candida* strains to antifungals and evaluate their role in drug response.

**Results:**

Herein, Chr4 was truncated in *C. albicans* using the telomere-mediated chromosomal truncation method. The resulting eight *Candida* strains carrying one truncated homolog of Chr4 were tested for response to multiple antifungals. The minimal inhibitory concentration (MIC) for these strains was determined against three classes of antifungals. The MIC values against fluconazole, amphotericin B, and caspofungin were closer to that of the wild type strain. Microdilution assay against fluconazole showed that the mutants and wild type strains had similar sensitivity to fluconazole. The disc diffusion assay against five azoles and two polyenes revealed that the zones of inhibition for all the eight strains were similar to those of the wild type. Thus, none of the generated strains showed any significant resistance to the tested antifungals. However, spot assay exhibited a reasonably high tolerance of a few generated strains with increasing concentrations of fluconazole.

**Conclusion:**

This analysis suggested that Chr4 aneuploidy might not underlie drug resistance but rather drug tolerance in *Candida albicans*.

**Supplementary Information:**

The online version contains supplementary material available at 10.1186/s43141-021-00197-0.

## Background

*Candida albicans* is a diploid polymorphic fungus that grows as yeast with pseudohyphae and true hyphae [1, 2]. It resides as a harmless commensal on the skin and in the mucosal lining of the gastrointestinal and genitourinary tracts in humans and is the most prevalent fungal pathogen that can cause both superficial (such as oral and vaginal candidiasis) and systemic infections [3, 4]. Systemic infections are life-threatening and common in immunologically weak individuals, such as HIV patients, neonates with low birth weight, transplant recipients, and chemotherapy patients [5]. Several other species of *Candida*, including *C. glabrata, C. tropicalis, C. parapsilosis*, *C. haemulonii*, and *C. krusei* were also isolated from clinical samples; however, *C. albicans* remains the most common fungal pathogen [6]. Several potential antifungals are extensively used in clinical settings for the treatment and management of *Candida* infections. Based on the chemical compositions, the antifungals are classified into several groups: polyenes, pyrimidine analogs, echinocandins, thiocarbamates, allylamines, azoles, and morpholines [7]. These drugs inhibit the biosynthesis of crucial molecules, such as ergosterol and β-1,3-glucan, which are essential components of the fungal cell. Some antifungals can perforate the cell wall leading to the death of the fungi [8, 9]. Intriguingly, the clinical isolates are becoming increasingly resistant to the available antifungals, which pose a threat to the treatment and management of *Candida* infections, especially bloodstream infections [10, 11].

The drug resistance in *C. albicans* has been studied extensively in the last two decades to comprehend the underlying mechanism. It seems to have developed drug resistance via overexpression of drug transporters, alterations of drug targets, utilization of compensatory and catabolic pathways, and biofilm formation [11–13]. However, the ABC (ATP-binding cassette) (Cdr1p, Cdr2p) and major facilitator superfamily (MFS) (Mdr1p) transporters are considered the major contributors to drug resistance in *C. albicans* [13, 14].

Moreover, the modifications or alterations of biosynthetic pathways significantly contribute to drug resistance in this fungal pathogen. For example, the azole drugs (including fluconazole) inhibit the enzyme 14-alpha lanosterol demethylase (encoded by the *ERG11* gene) required for the biosynthesis of ergosterol. However, multiple mutations in the *ERG11* gene (encoding lanosterol 14α-demethylase) make inactive these drugs against *C. albicans* [15]. In addition, mutations in *ERG3* (encoding sterol Δ^5,6^ desaturase) render resistance to *Candida* cells as the mutated enzyme fails to convert 14α-methylated sterols into toxic 3,6-diol derivatives [16]. Moreover, the effect of the toxic compound, 5-fluorocytosine (5-FC), is nullified by incorporating the mutations in the *FUR1* gene encoding uracil phosphoribosyltransferase [17]. The mutations in the *FSK1* gene encoding a subunit of β-1,3-glucan synthase complex render the *Candida* cells resistant to echinocandins [18]. In addition, many azole-resistant clinical isolates of *C. albicans* acquired gain-of-function mutations in the transcription factors: Tac1p, Mrr1p, and Upc2p [19, 20], which can counteract environmental stress and survive in adverse conditions.

Furthermore, pathogenic yeast *C. albicans* chromosomal nondisjunction causes genetic changes in response to environmental cues [21, 22]. This phenomenon generates aneuploidies that can adapt to stressful conditions for survival. In the presence of fluconazole, nondisjunction is observed in *C. albicans*, giving rise to Chr4 monosomy and Chr3 trisomy. The strains bearing these alterations become resistant to fluconazole [23]. Interestingly, in the absence of a complete sexual cycle, *C. albicans* resorts to a parasexual cycle in which two diploid cells generate a tetraploid. Subsequently, the tetraploids undergo a concerted chromosome loss and generate multiple aneuploidies, i.e., it can generate numerous changes in the genome, including but not limited to genome organization and variations in chromosome copy number. The *Candida* cells grown under high concentrations of fluconazole can incorporate numerous changes in the whole genome [23]. Therefore, the presence of Chr4 monosomy in fluconazole-resistant mutants might not confer fluconazole resistance.

Hence, a systematic chromosomal truncation approach should be applied to validate Chr4 monosomy and its relation to fluconazole resistance. This method was successfully applied to understand the regulation of L-sorbose utilization in *C. albicans* [24, 25]. Therefore, in this study, we adopted the telomere-mediated chromosomal truncation method [24] to truncate Chr4 and assess the response of the isoform against antifungals. Herein, we have carried out eight systematic truncations in one of the homologs of Chr4, whereas the second homolog remains intact. Next, the strains carrying the truncated Chr4 were tested against three classes of commonly used antifungals: azoles, polyenes, and echinocandins. This systematic study suggested that Chr4 may not be involved in drug resistance. However, it could play an essential role in the drug tolerance of this pathogen.

## Methods

### Strains, media, and growth conditions

*C. albicans* strain CAF4-2 (∆*ura3::imm434*/∆*ura3::imm434*) was used for chromosomal truncations [26]. The yeast *Saccharomyces cerevisiae* strain B-8728 (*MAT*a *ura3-52 trp1-*Δ*63 leu2-*Δ*1 GAL2*) was used to prepare the chromosomal size marker (obtained from Fred Sherman, University of Rochester, USA). *Escherichia coli* strain XL-1 Blue was used for creating the plasmid constructs and routine amplification of plasmids [27].

Yeast extract/peptone/dextrose (YPD) and synthetic media were prepared as described previously [28]. Uridine was added at a concentration of 50 μg/mL to the media as required. All the strains were routinely grown at 30 °C. Fluconazole (Sigma, USA) solution (10 mg/mL) was prepared in dimethyl sulfoxide (DMSO). The bacterial strains were grown in YT media (0.5% sodium chloride, 0.5% yeast extract, and 1% tryptone) at 37 °C. The *E. coli* strains containing plasmids were grown in YT media containing 100 μg/mL ampicillin. The stock solution of zymolyase (Sigma) (1 mg/mL) was prepared in 15% glycerol and stored at − 20 °C. Mueller-Hinton agar plates (Himedia, Mumbai, India) were used for minimum inhibition concentration (MIC) and disc diffusion assays.

### Molecular biology methods

Molecular biology methods, including restriction digestion of plasmids, gel elution of DNA fragments, ligation of DNA fragments into vectors, and polymerase chain reaction (PCR), were carried out as described previously [29]. Plasmids from *E. coli* were isolated using the alkaline lysis method [30]. *E. coli* transformation was carried out using the calcium chloride method [29], and *C. albicans* transformation was performed using the spheroplast method [31, 32].

### Vectors and plasmid constructions

The pSFU1 plasmid containing a *URA3* flipper was a kind gift from J. Morshhauser [33]. The plasmid “BSA” containing *C. albicans* telomere was obtained from McEachern and Hicks [34]. The plasmids pRC3915 (integrative plasmid) and pRC2312 (replicative plasmid) were obtained from Cannon et al. [35]. The plasmid pKA05 was constructed by subcloning a 0.75-kbp *Sal*I-*Sac*I fragment containing *Candida* telomere from the plasmid “BSA” into pSFU1 at the *Not*I-*Sac*I site as described previously [25] and used as the basic plasmid for generating truncation constructs (Supplementary Fig. S1A). For truncating Chr4 at the intended sites, the DNA sequences were retrieved from the *Candida* Genome Database (CGD) [36], and the primers were designed (Table 1) to amplify the mapping sequence (MS). The PCR products were first cloned into pTZ57R/T vector using InsTA PCR Cloning kit (Thermo Fisher Scientific, Vilnius, Lithuania) and subsequently into pKA05 to generate the truncation constructs. The plasmid pKA484 was constructed by inserting a 2.2-kbp *Pst*I-*Bam*HI fragment (containing the open reading frame *Orf19.3120*) into the vector pRC2312. This construct was used for overexpression of *Orf19.3120*. The plasmids used in this study are listed in Table 2.

**Table 1:** Primers used in this study

Name	Sequence (5′ to 3′)	Truncation sites on Chr4
**For truncation constructs**		
KC32	TCG CTC GAG ACT TGG GAT AAG GAG AGC AAA	969.925 (1)
KC33	TCG GGT ACC TGT TGC TGA CGA TGT TGA	
KC34	TCG GGT ACC ATT TGT ACT GTT TTG CGT CTG	1002.852 (2)
KC35	TCG CTC GAG GAC TCA CCA ATA GTT CAA GGC A	
KC432	GGA TCC CTG CTA AAC GAT ACC AGC AAT TAA CT	1102.087 (3)
KC433	GAG CTC TGT GTC CAT CAA AGC CCA AT	
KC434	GGA TCC GCA CAA CCA ATT GAA GCT GGT A	1201.989 (4)
KC435	GAG CTC CGT TTC TAA CAG TTC TTG CAC G	
KC436	GGA TCC CAG ACG ATA AGT GAA TAT CTC G	1301.783 (5)
KC437	GAG CTC GGA ATA CTA TGT GTG TCA AGG GGA	
KC136	TCG GGT ACC AAT CAC CAT CCG ACG AGT ACT T	1369.883 (6)
KC137	TCG CTC GAG TCA TGT GCT TTT GTC TC	
KC139	TCG GGT ACC TAG AAT GGC CAT GTT GCA TAG TC	1529.969 (7)
KC140	TCG CTC GAG CAC ATC GTA CCG TAA TTG AAA	
KC172	GGT ACC GAT CTT GCT TTG TTC CTA CAA A	1542.907 (8)
KC173	CTC GAG CCA AAG CAA CAG CCG AAT ACT A	
**For verification of truncation junctions**		
KC49	CCA GCA AGA CTT TGC AAT GT	969.925
KC48	GAT TTA AAG CTC CAT GTG CCA	1002.852
KC442	CTG GCA ATG AAC GAT CAA GT	1102.087
KC443	ACC AGC ATG ATA GAA CCC AA	1201.989
KC444	CAA GAT AGC TAG AGC TCA GA	
KC445	AGT CCT ACG TAG GTC AGT AA	1301.783
KC138	TCC AAG AAA CAA TTT GAA AAT CCA	1369.883
KC141	CCA CAA ATC AAT TGC AAA C	1529.969
KC174	TTC GTT CGA TGC TCG TAT TCT AG	1542.907
KC27	TTG ATG CAT TAA ACA CCT TGA	*URA3* flipper
KC102	TCC TAT TCC TTC TCC TTA TGG C

**Table 2:** List of the plasmids used in this study

Sl. No.	Plasmid	Description	References
1.	pSFU1	Vector	[33]
2.	“BSA”	*Candida* telomere	[34]
3.	pRC3915	Vector	[35]
4.	pRC 2312	Vector	[35]
5.	pKA05	Backbone plasmid	[25]
6.	pKA52	Cassette for truncation 1	This study
7.	pKA45	Cassette for truncation 2	This study
8.	pKA627	Cassette for truncation 3	This study
9.	pKA628	Cassette for truncation 4	This study
10.	pKA629	Cassette for truncation 5	This study
11.	pKA154	Cassette for truncation 6	This study
12.	pKA163	Cassette for truncation 7	This study
13.	pKA191	Cassette for truncation 8	This study
14.	pKA484	Overexpression for *Orf19.3120*	This study

### Chromosomal preparation, separation, and band analysis

In order to verify the karyotypes of the generated *Candida* strains, intact chromosomes were prepared as described previously [32]. Briefly, fresh *C. albicans* cells were grown in YPD liquid media to the cell density of 10^7^ cells/mL and harvested by centrifugation. Cells were washed with 50 mM ethylenediaminetetraacetic acid (EDTA) pH 7.5 and resuspended at 1 × 10^9^ cells/mL. For making the plugs, 200 μL cells, 20 μL zymolyase, and 250 μL of 1.2% low-melting agarose were mixed vigorously in a 1.5-mL tube and poured into the molds (Bio-Rad Laboratories, USA) as 100 μL aliquots/well and solidified at room temperature for 20 min. First incubation was at 37 °C for 18 h in solution containing 0.5 M EDTA (pH 9.0), 25 mM Tris-HCl (pH 8.0), and 4% β-mercaptoethanol, and the second incubation was at 55 °C in 0.5 M EDTA (pH 9.0), 25 mM Tris-HCl (pH 8.0), 1% N-lauroylsarcosine, and 0.01% proteinase K for 48 h.

The pulse-field gel electrophoresis system CHEF-DR II (Bio-Rad) was used in separating the chromosomes as described previously [32]. Agarose gels were stained using SYBR Green I nucleic acid gel stain (Sigma) for 30 min. The images of the gels were analyzed using Quantity One Software, and the intensity of the chromosome bands was analyzed by Image Lab Software Version 5.1 (Bio-Rad).

### Minimum inhibitory concentration (MIC) assay

The MIC for the generated *Candida* strains was determined according to the protocol of the Clinical and Laboratory Standards Institute (CLSI) [37]. Briefly, fresh cells were collected from YPD plates and counted under a light microscope. Approximately 2 × 10^6^ cells were mixed with 0.7% molten agar and poured onto Mueller-Hinton agar plates. The MIC strips were placed in the middle of the plate and incubated at 30 °C for 24 h. The images were captured using Quantity One Software. Ezy MIC^TM^ strips were used for three classes of antifungals: fluconazole (0.016–256 μg/mL), amphotericin B (0.002–32 μg/mL), and caspofungin (0.002–32 μg/mL) (Himedia, Mumbai, India).

### Spot assay against fluconazole

For spot assay on fluconazole plate, young cells were collected from YPD plates streaked directly from − 80 °C. The cells were counted under a light microscope and spotted on synthetic dextrose (SD) plates containing fluconazole as 10-fold serial dilutions.

### Microdilution assay against fluconazole

We carried out broth microdilution assay against fluconazole as described previously [37, 38]. The drug sensitivity test was carried out for the strains carrying truncations in one of the homologs of chromosome 4. Briefly, young cells were collected from the YPD plate, washed, and counted under a light microscope. Then, approximately 200 cells/well were inoculated into 96-well flat-bottomed microtiter plate. Subsequently, the antifungal drug fluconazole was added to the wells in increasing concentration from 0 to 128 μg/mL. The wells containing only media and media plus cells (without drug) were considered negative and positive controls. All the samples were plated in triplicate and incubated at 30 °C for 24 h, and optical densities (OD) were measured at 600 nm using a multi-detection microplate reader (Thermo Fisher Scientific, USA). Then, the graph of OD_600_ vs. fluconazole concentrations.

### Disc diffusion assay

The disc diffusion test for *Candida* strains was carried out as described previously [39]. Discs containing fluconazole (25 μg), itraconazole (10 μg), ketoconazole (10 μg), clotrimazole (10 μg), miconazole (30 μg), amphotericin B (50 μg), and nystatin (50 μg) were used for disc diffusion assay (Himedia). Approximately 2 × 10^6^ cells were mixed with 0.7% molten agar and poured onto Mueller-Hinton agar plates. The agar solidified for 15 min, and then the discs containing antifungals were placed in the middle of the plate. Next, we incubated the plates at 30 °C and measured the zone of inhibition at 24 h.

## Results

### Truncations of Chr4 in *C. albicans*

**Fig. 1:**
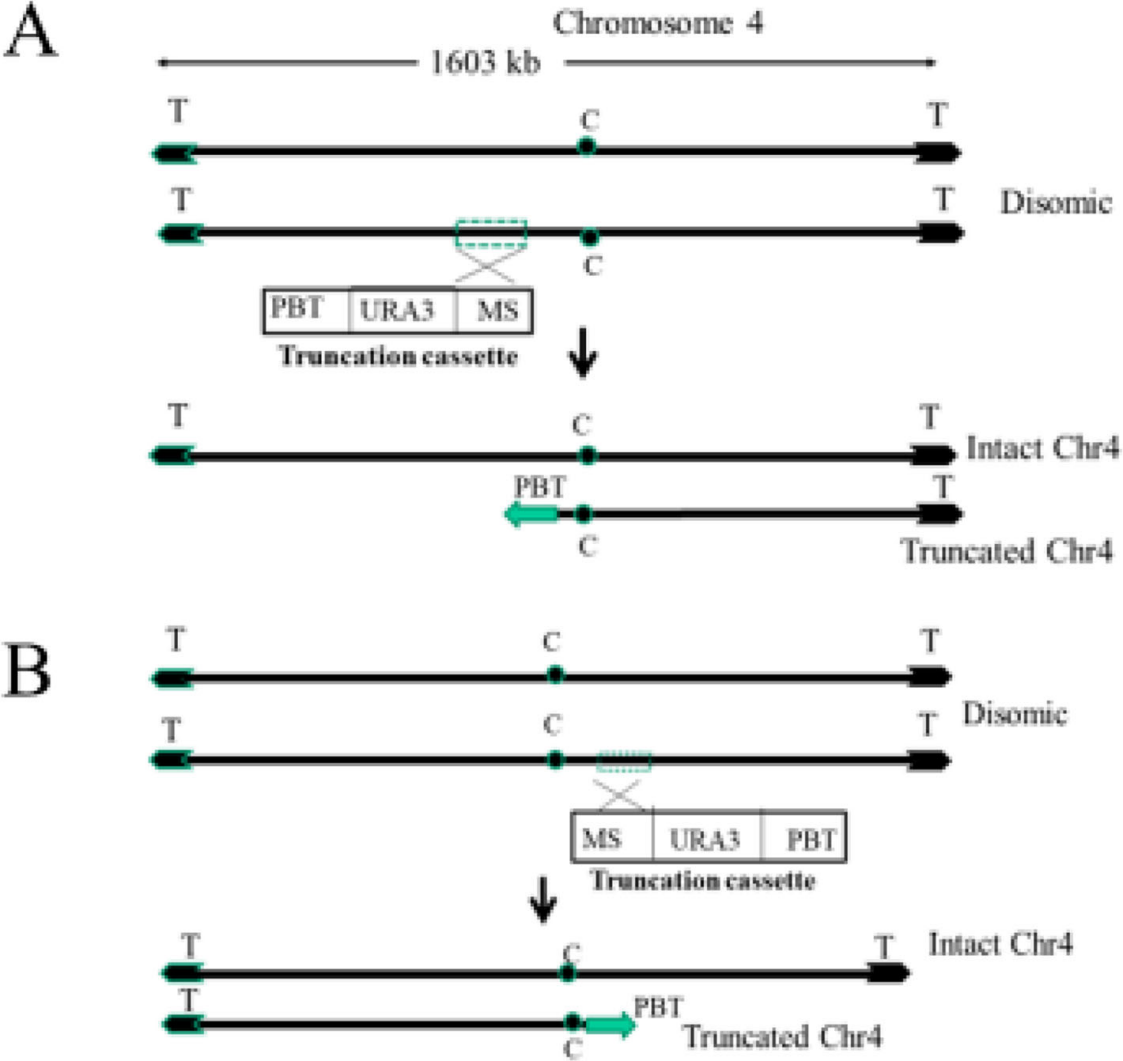
Schematic diagram of chromosomal truncation. **A** Truncation on the left side of the centromere of chromosome 4. Two thick lines represent two homologs of Chr4 containing a centromere (C) in the middle and two telomeres at the ends (T). Truncation cassette has three essential components: PBT, plasmid-borne telomere sequence cloned in the same orientation as it is present in the cellular chromosome; MS, mapping sequence retrieved from the chromosomal sequence at which truncation is intended; and *URA3*, selection marker. **B** Truncation of Chr4 on the right-hand side of centromere. Truncation cassettes are made similarly to left portion truncation. However, the telomere sequence will face towards the right and will replace the cellular right portion telomere. Truncation on either side of the centromere generates a truncated homolog of Chr4 wherein the second homolog remains intact

**Fig. 2:**
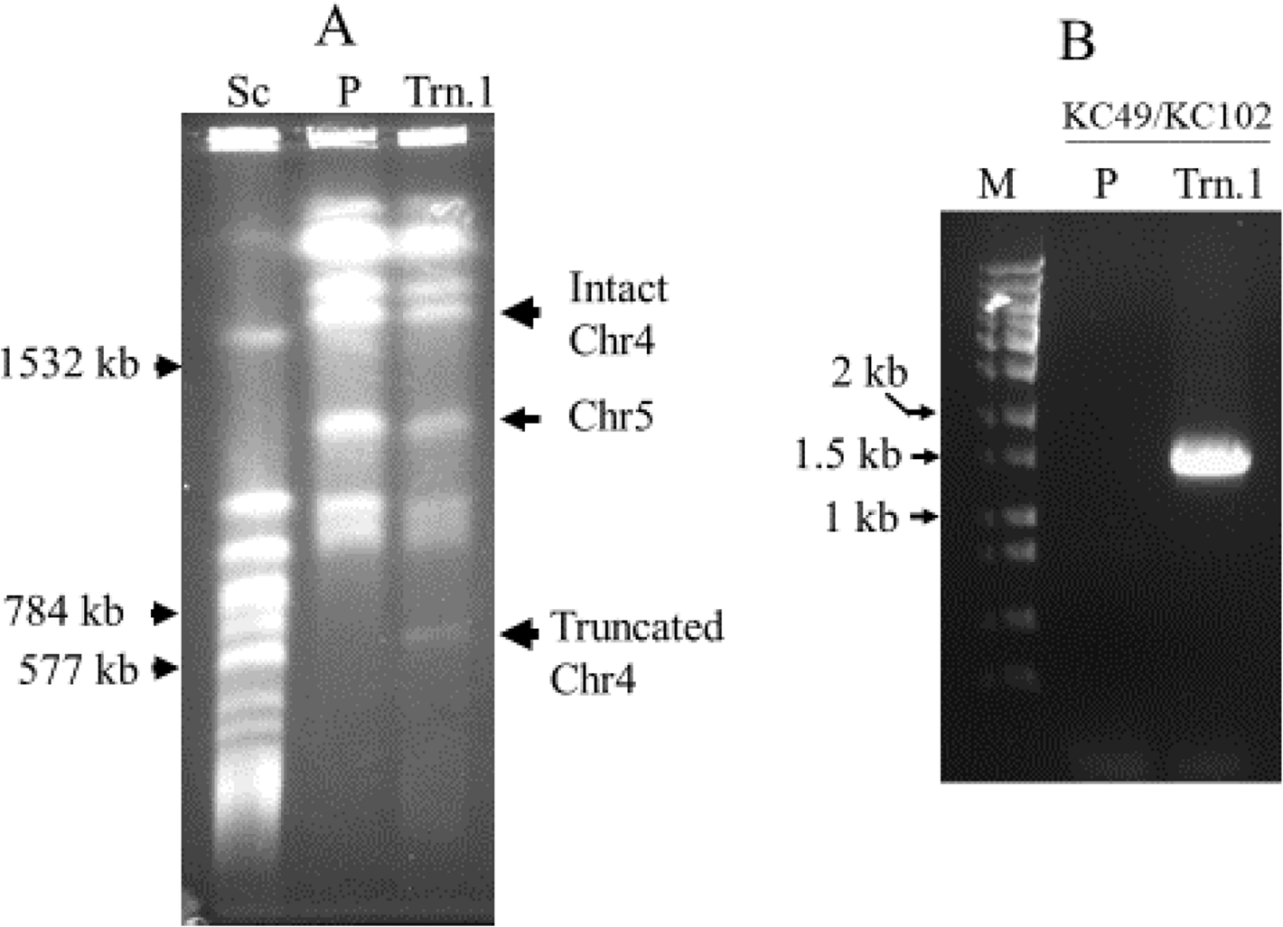
Verification of truncation 1. **A** Karyotypic verification of truncation 1 (969.925 kb position) of chromosome 4 using contour-clamped homogenous electric field (CHEF). Sc, *Saccharomyces cerevisiae* chromosomes used as size marker, three relevant chromosome sizes are indicated by arrows at 1532 kb, 784 kb and 577 kb respectively; P, wild type strain CAF4-2; Trn.1, chromosomes of the strain carrying truncation 1. Arrows indicated for the intact homolog and truncated version of Chr4. Truncation at 969.925 kb position (~ 970 kb) generates a 633 kb long truncated version of Chr4. It runs between 784 kb and 577 kb marker chromosomes of *S. cerevisiae*. **B** PCR verification of truncation 1 with specific primers KC49/KC102. Lane M, 1 kb DNA ladder; lane P, PCR for wild type strain (no PCR product amplified); lane Trn.1, PCR for truncation junction producing 1.5 kb PCR product (expected size)

In order to elucidate the role of Chr 4 concerning drug resistance, we adopted a well-established telomere-mediated chromosomal truncation approach that analyzes L-sorbose utilization in this pathogen [24, 25]. To truncate Chr4, approximately 1 kbp PCR product (MS) was amplified from the intended truncation site using genomic DNA of parental strain CAF4-2, and then the truncation cassette was constructed. The exogenous construct consisted of three essential components: MS, selection marker (*URA3*), and *Candida* telomere (plasmid-borne telomere, PBT). After transformation into *Candida* strain, the truncation cassette replaces the entire portion of one homolog of Chr4 from the intended site to the telomere (Fig. 1). Furthermore, Chr4 is 1603 kbp long in which the centromere is located at 992–996.2 kbp position according to assembly 22 of the *Candida* Genome Database [36]. Since the left portion of Chr4 is longer (~ 992 kbp) than the right portion (~ 606 kbp), we performed the first truncation (truncation 1) at 969.925 kbp position (left side of the centromere) to remove approximately 970 kbp of the left portion as shown in Fig. 1a. The plasmid pKA52 was digested with *Kpn*I-*Sac*I to release the truncation cassette, which was transformed with the wild type strain CAF4-2. The ura^+^ transformants were screened by PCR using specific primers KC49/KC102 to identify the appropriate candidates for this truncation (Table 1, Supplementary Fig. 1B). Subsequently, we verified the truncated homolog of Chr4 by running a contour-clamped homogenous electric field (CHEF) program; nine positive candidates were obtained (Table 3). The chromosomal separation of one representative candidate of truncation 1 (969.925 kbp position) is shown in Fig. 2a. The size of the intact homolog of Chr4 is 1603 kbp, which is slightly above the 1532 kbp size marker of *S. cerevisiae*. However, the expected size of the truncated homolog of Chr4 was approximately 638 kbp (including *URA3* flipper) and between 577 kbp and 784 kbp of *S. cerevisiae* markers (Fig. 2a). The PCR verification results for truncation 1 are shown in Fig. 2b. The primers KC49/KC102 could amplify the 1.5 kbp PCR product if truncation occurs at 969.925 kbp position (truncation1). The *Candida* strain carrying truncated Chr4 (truncation 1) showed a 1.5 kbp band on agarose gel electrophoreses, whereas wild type strain (no truncation) did not produce any PCR product (Fig. 2b).

**Table 3:** Candidates obtained after PCR screening and CHEF verification

Truncation number	Position on Chr4 (kb)	Number of right candidates^a^
Trn.1	969.925	9
Trn.2	1002.852	15
Trn.3	1102.087	13
Trn.4	1201.989	5
Trn.5	1301.783	2
Trn.6	1369.883	3
Trn.7	1529.969	16
Trn.8	1542.907	12

^a^Approximately 40–50 ura^+^ transformants screened for each truncation

**Fig. 3:**
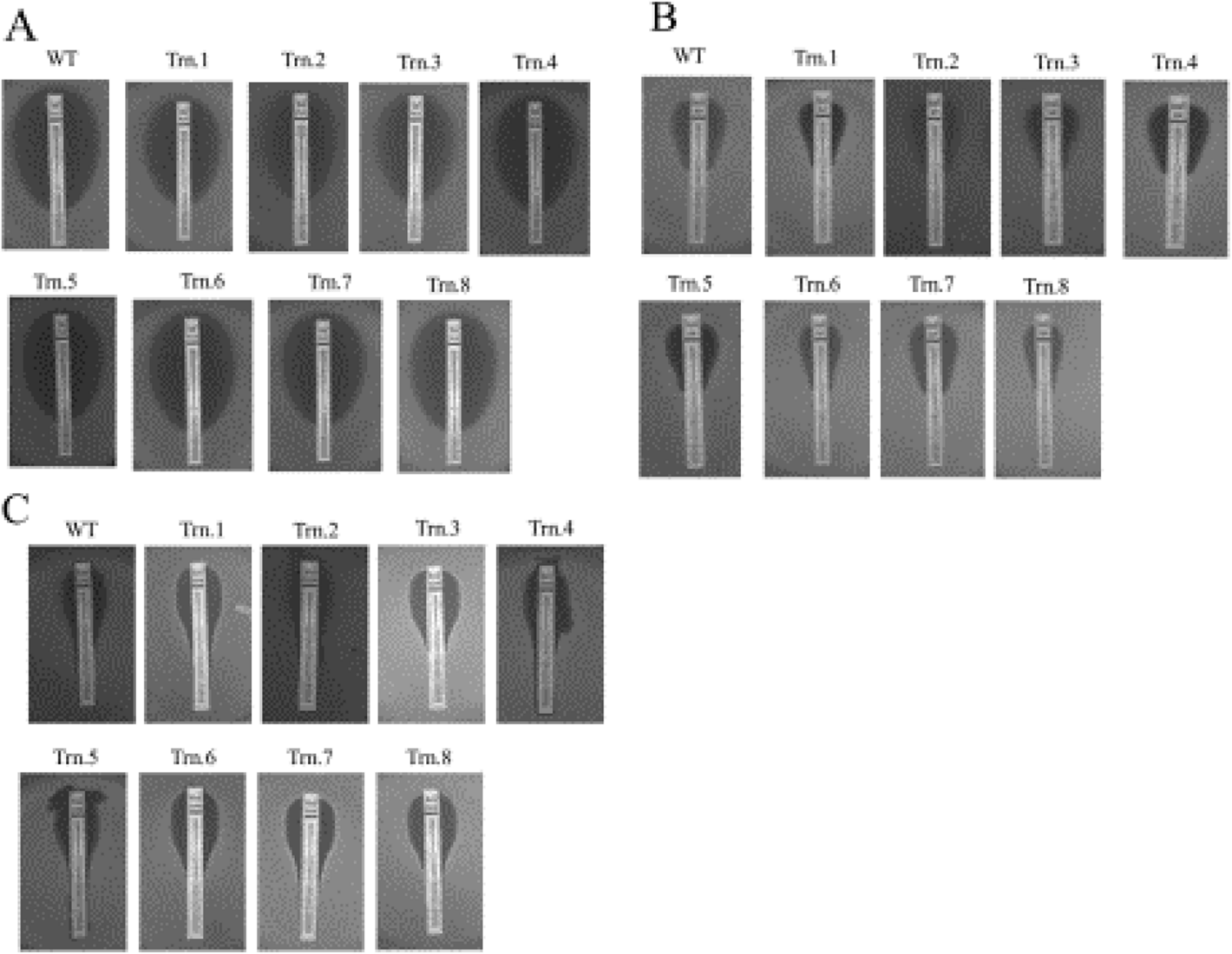
Minimum inhibitory concentration (MIC) assay for strains carrying truncated Chr4. WT, wild type strain CAF4-2; Trn.1 to Trn.8, *Candida* strains carrying truncations 1–8. MIC strip test for the strains against **A** fluconazole in the range of 0.016–256 μg/ml; **B** amphotericin B in the range of 0.002–32 μg/ml; **C** caspofungin in the range of 0.002–32 μg/ml. Strains with truncated Chr4 showed MIC values similar to the wild type strain

To determine whether the *Candida* strain carrying truncation 1 becomes resistant, we conducted the MIC test with three antifungal classes: fluconazole (azole), amphotericin B (polyene), and caspofungin (echinocandin). The MIC values for truncation 1 (at 969.925 kbp position) against fluconazole, amphotericin B, and caspofungin were 0.25, 1.0, and 0.125 μg/mL, respectively, whereas those for the wild type strain were 0.19, 0.75, and 0.064 μg/mL, respectively. These findings suggested that the strain carrying the truncated version of Chr4 (truncation 1) is sensitive to these antifungals, similar to that observed for the wild type strain (truncation 1 in Fig. 3, Supplementary Table S1).

**Fig. 4:**
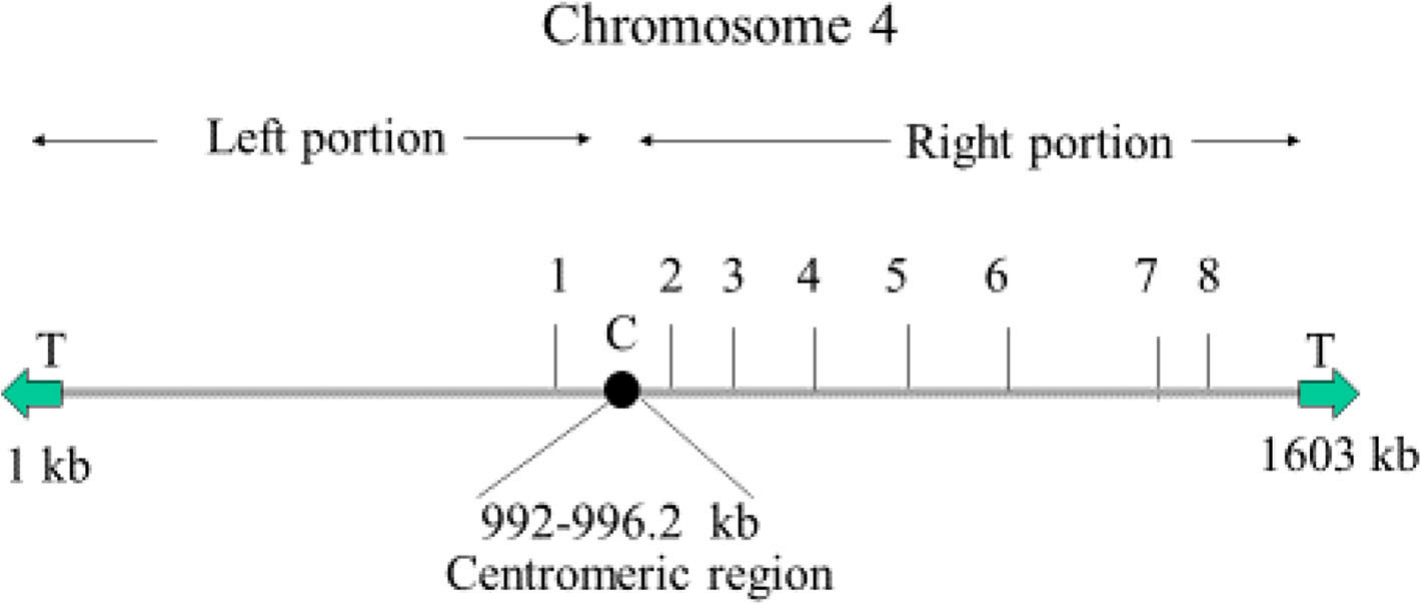
The schematic diagram of eight truncations on chromosome 4 (1603 kb long). Truncation 1 at 969.925 kb position on the left side of the centromere. Truncations 2–8 at 1002.852 kb, 1102.087 kb, 1201.989 kb, 1301.783 kb, 1369.883 kb, 1529.969 kb, and 1542.907 kb positions on the right portion of Chr4. The centromere spanned at 992–996.2 kb position according to *Candida* Genome Database

**Fig. 5:**
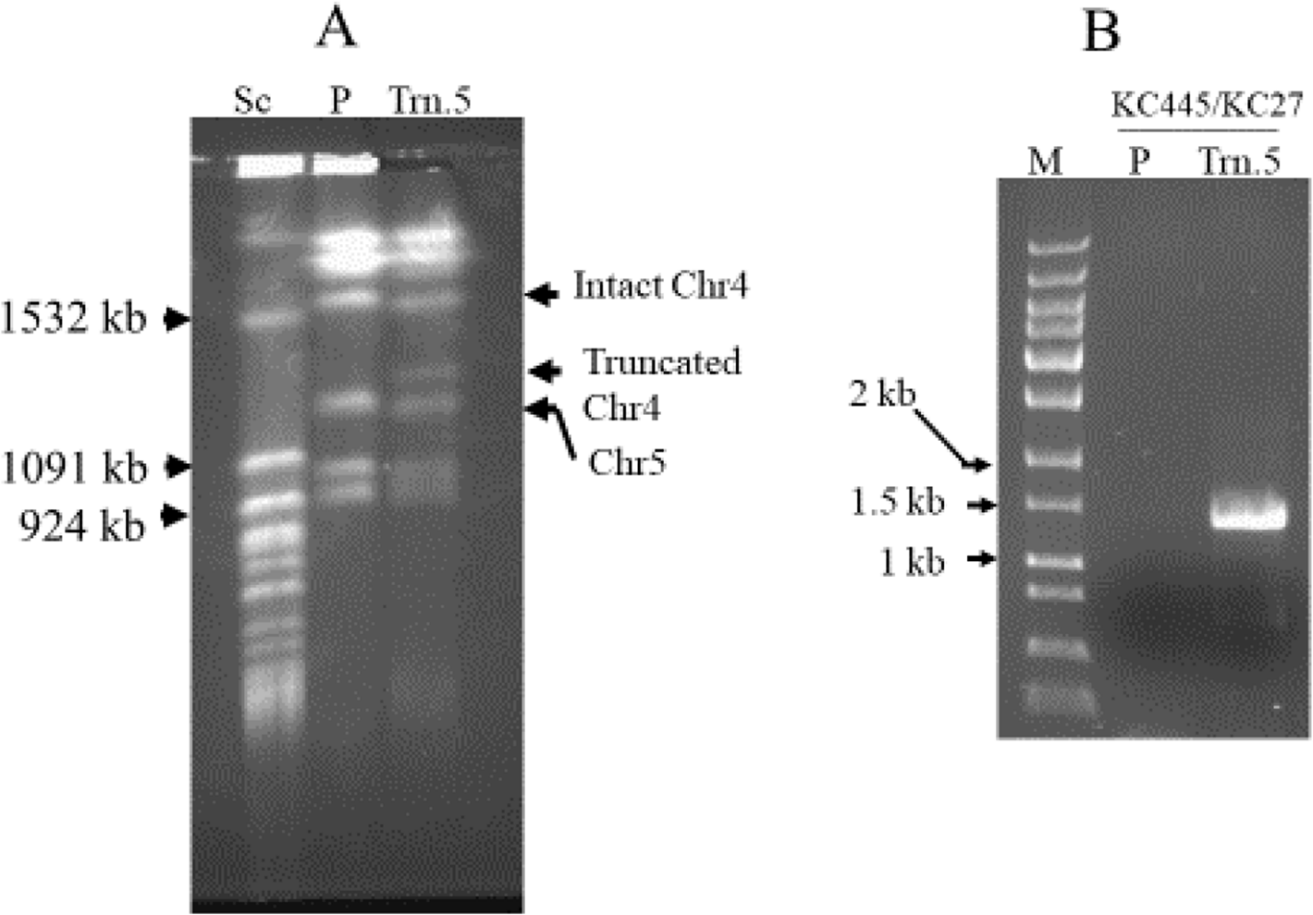
Verification of truncation 5 on chromosome 4. **A** Karyotypic verification using CHEF. Sc, *S. cerevisiae* chromosomes size marker, three relevant chromosome sizes are indicated by arrows at 1532 kb, 1091 kb, and 924 kb respectively; P, wild type strain CAF4-2; Trn.5, chromosomes of the strain carrying truncation 5 at 1301.783 kb position on Chr4. The size of the truncated version of Chr4 (~ 1305 kb) is approximately 300 kb shorter than intact Chr4 and runs above Chr5 (Chr5 size---1191 kb). **B** PCR verification of truncation 5 with specific primers KC445/KC27. Lane M, 1 kb DNA ladder; lane P, PCR for wild type strain (no PCR product amplified); lane Trn.5, PCR for truncation junction producing 1.5 kb PCR product (expected size)

As the removal of the left portion of Chr4 failed to produce any resistant phenotype, we carried out seven additional truncations (2–8) on the right portion of Chr4. These seven truncations were performed at 1002.852, 1102.087, 1201.989, 1301.783, 1369.883, 1529.969, and 1542.907 kbp positons on the right portion of Chr4 (Fig. 4). The karyotype of one representative candidate of truncation 5 (1301.783 kbp position) is shown in Fig. 5a. The truncated homolog of Chr4 (approximately 1305 kbp) runs just above Chr5 (size of Chr5 is 1191 kbp) and below the 1532 kbp size marker of *S. cerevisiae*; this truncation was further validated by PCR (Fig. 5b). Moreover, we attempted to truncate both left and right portions of Chr4 in the same strain to mimic Chr4 monosomy. However, we did not obtain any candidate after screening approximately 100 transformants. This failure could be attributed to two compelling reasons: First, the generation of monosomy of Chr4 may be a low-frequency event in *C. albicans*, and second, the deletion of both the left and right portions of Chr4 could be lethal due to the presence of recessive mutations or allelic differences.

In summary, we performed eight systematic chromosomal truncations in one of the two homologs of Chr4 (Table 3). Truncations 1 and 2 removed the left and right portions of Chr4, respectively. Five truncations on the right portion of Chr4 (truncations 2–6) were performed at about 100 kbp from each other. On the other hand, the distance between truncations 7 and 8 was about 13 kbp from each other and was carried out to assess the role of the open reading frame (ORF) *Orf19.3120* in drug resistance; it encodes a half-size PDR-subfamily ABC (ATP-binding cassette) transporter [36, 40].

### Determination of MIC values for the generated strains

We determined the MIC values for all the eight *Candida* strains carrying a truncated version of one homolog of Chr4. These strains were tested using MIC strips for fluconazole, amphotericin B, and caspofungin (Fig. 3, Supplementary Table S1). The MIC values for fluconazole, amphotericin B, and caspofungin were as follows: 0.19–0.38 μg/mL (wild type 0.19 μg/mL), 0.75–1.0 μg/mL (wild type 0.75 μg/mL), and 0.064–0.25 μg/mL (wild type 0.064 μg/mL), respectively. These MIC values indicated that the strains bearing the truncated version of Chr4 are not drug-resistant.

### Microdilution assay against fluconazole

**Fig. 6:**
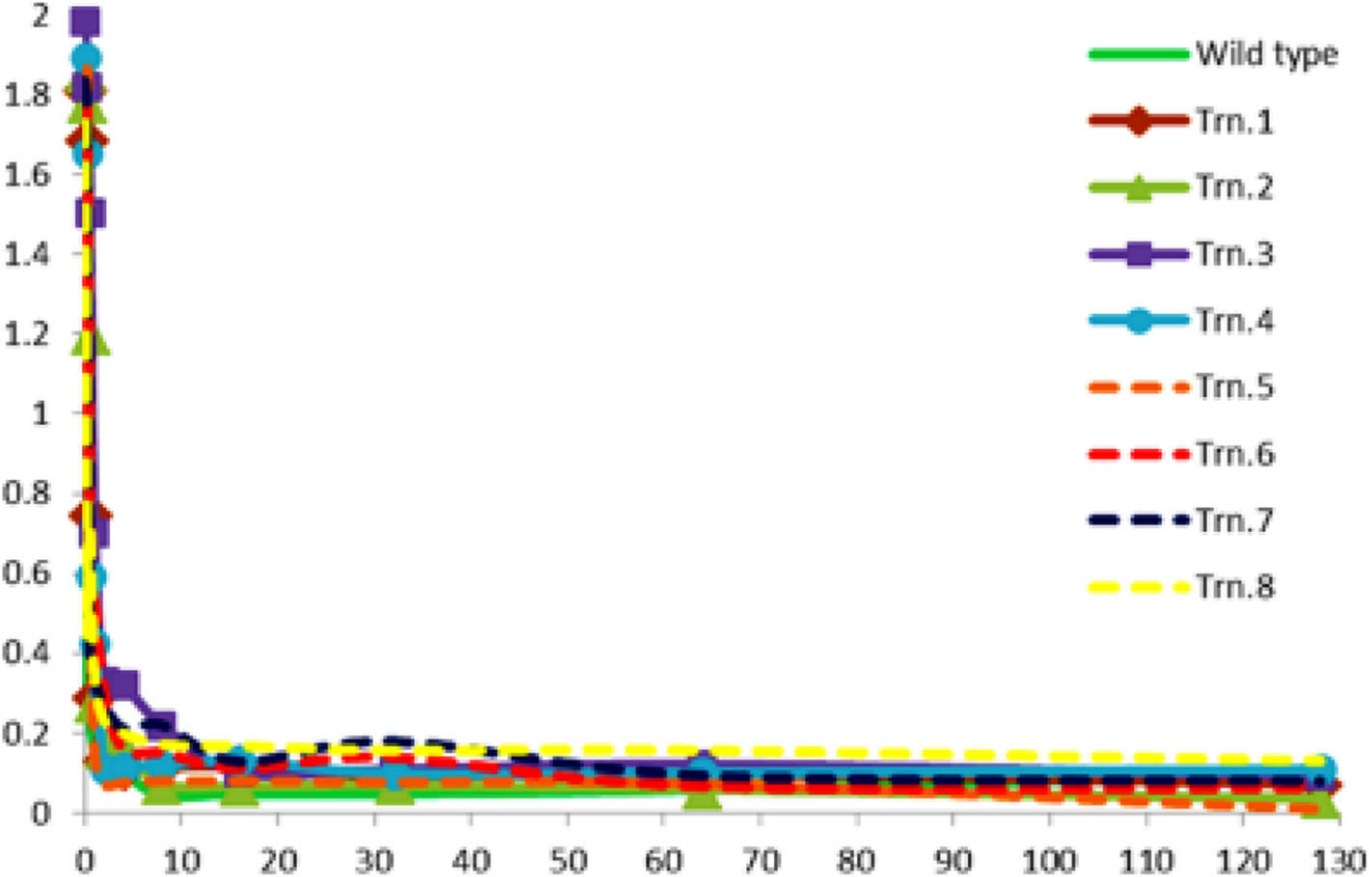
Broth microdilution assay of the generated strains against fluconazole. WT, wild type strain CAF4-2; Trn.1 to Trn.8, *Candida* strains carrying truncations 1–8. Fluconazole concentrations from 0 to 128 μg/ml (0, 0.25, 0.5, 1, 2, 4, 8, 16, 32, 64, and 128 μg/ml) were used. All the strains are showing sensitivity to fluconazole, similar to the wild type strain

We carried out a microdilution assay against fluconazole for all the eight strains carrying truncations in one homolog of Chr4. The *Candida* strain CAF4-2 transformed with the integrative plasmid pRC3915 served as a control. Readings were recorded 24 h after inoculation, and graphs were plotted against fluconazole concentrations (Fig. 6). These results demonstrated that the generated strains remain sensitive to fluconazole.

### Spot assay against fluconazole

**Fig. 7:**
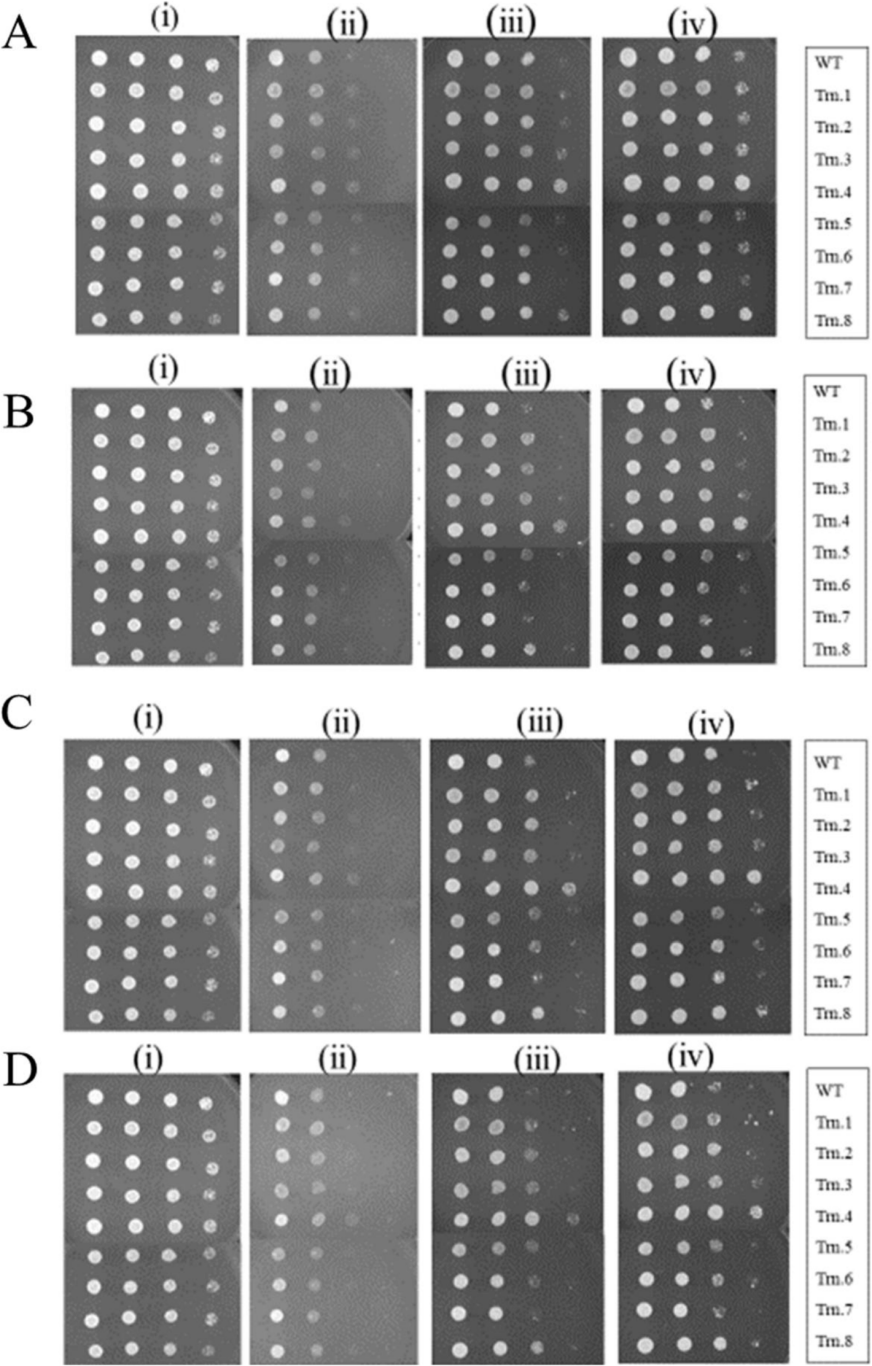
Spot assay against fluconazole for the strains carrying truncated Chr4. WT, wild type strain CAF4-2; Trn.1 to Trn.8, *Candida* strains carrying truncations 1–8. Strains were 10-fold serially diluted (starting with 10^7^/ml) and spotted on plate containing fluconazole **A** 8 μg/ml, **B** 16 μg/ml, **C** 32 μg/ml, and **D** 64 μg/ml where (i) control plate without fluconazole and photograph was taken after 24 h. For a plate containing fluconazole, we took photographs at 24 h (ii), 48 h (iii), and 72 h (iv) after spotting

We spotted *Candida* strains carrying truncated homolog of Chr4 on SD plates containing various concentrations of fluconazole: 8, 16, 32, and 64 μg/mL (starting with 10^7^ cells/mL). We considered high concentrations of fluconazole due to its fungistatic nature. The plates were incubated at 30 °C, and images were captured every 24 h for 3 days. The spotting assay of the generated strains on fluconazole showed distinct phenotypes when incubated for a prolonged period. The strain-sensitivity to fluconazole in 24 h was similar to that in the wild type strain; however, differential sensitivity towards fluconazole was detected in 48 h, which became prominent in 72 h (Fig. 7). The strain carrying truncation 5 was most sensitive among the strains. However, the strain carrying truncation 4 showed the least sensitivity to fluconazole, followed by truncation 8, while the other five stains did not differ markedly compared to the wild type strain. This analysis suggested that some truncated homologs of Chr4 were tolerant to fluconazole, although the MIC values were in the sensitive range.

### Disc diffusion assay

We conducted a disc diffusion assay for eight strains against seven antifungals, including five azoles and two polyenes. All the strains showed sensitivity to these drugs, forming a clear zone of inhibition (Table 4, supplementary Fig. S2). However, the diameter of the zone of inhibition varied among the strains. The diameters of the zone of inhibition for the strains 32–39 mm against fluconazole (wild type, 37 mm), 23–32 mm against clotrimazole (wild type, 27 mm), 22–33 mm against itraconazole (wild type, 29 mm), 23–38 mm against ketoconazole (wild type, 34 mm), 25–41 mm against miconazole (wild type, 34 mm), 17–25 mm against amphotericin B (wild type, 24 mm), and 19–32 mm against nystatin (wild type, 30 mm). The variation in the drug sensitivity could be attributed to the strains’ genetic makeup as Chr4 was truncated at different sites removing large chromosomal segments of various sizes.

**Table 4:** Zone of Inhibition diameter for different drugs (mm)

Truncations	Truncation sites	Drugs and diameter (mm)^a^						
		FLC	CC	IT	KT	MIC	AP	NS
WT	Intact Chr4	37	27	29	34	34	24	30
Trn.1	969.925	33	29	25	28	36	17	22
Trn.2	1002.852	32	23	22	33	25	25	30
Trn.3	1102.087	36	31	29	28	36	20	25
Trn.4	1201.989	39	32	33	38	36	23	30
Trn.5	1301.783	34	30	28	36	34	23	27
Trn.6	1369.883	37	32	25	23	34	20	19
Trn.7	1529.969	33	26	26	25	41	19	21
Trn.8	1542.907	36	29	27	29	35	18	20

^a^*FLC* fluconazole, *CC* clotrimazole, *IT* itraconazole, *KT* ketoconazole, *MIC* miconazole, *AP* amphotericin B, *NS* nystatin

### Overexpression of *Orf19.3120*

**Fig. 8:**
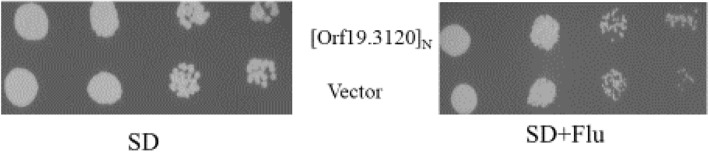
Overexpression of *Orf19.3120* and its response to fluconazole. Strains were 10-fold serially diluted and spotted on synthetic dextrose (SD), and synthetic dextrose plus fluconazole (8μg/ml) (SD+Flu) plates lacking uracil. The overexpressed strain grows a little better than the control strain in the presence of fluconazole

The overexpression of many genes renders the *Candida* strains resistant to multiple antifungals. Since ORF *Orf19.3120* was predicted to encode a half-size PDR-subfamily ABC transporter [40], we overexpressed the molecule in the CAF4-2 *Candida* strain to test its response to fluconazole. Thus, we transformed CAF4-2 with the plasmid pKA484 (*Orf19.3120* cloned in pRC2312). The transformants were detected on a fluconazole plate incubated at 30 °C for 2 days. Consequently, we found that the overexpression of *Orf19.3120* does not render the strain resistant to fluconazole. However, it grew slightly more than the control strain, indicating a putative role in fluconazole resistance (Fig. 8).

## Discussion

In the history of *C. albicans*, the role of aneuploidy formation exhibited an efficient and effective means to generate critical genome modifications in response to environmental cues [41]. These changes are critical, such as altered chromosome copy number and translocation of a segment or truncation of a chromosome that exhibits multiple phenotypes, including drug resistance. In this study, we adopted a systematic chromosomal truncation approach to truncate Chr4 and assessed the strains for antifungals responses. The eight chromosomal truncations on one homolog of Chr4 generated *Candida* strains that were tested against antifungals. Either of the two homologs of Chr4 has the same probability of truncation as both are the same except for single nucleotide polymorphisms (SNPs) [42]. Truncation in one homolog generates monosomy of Chr4 with respect to the portion removed. For example, truncation at 969.925 kbp position on the left portion leaves only one left portion of the second homolog giving rise to the strain monosomy of Chr4 with respect to the left portion. The removal of the left portions of both homologs is not possible as there is one essential gene in approximately every 16 kbp of chromosomal stretch [36].

The generated *Candida* strains carrying the truncated homolog of Chr4 were assessed against multiple antifungals using different methods, such as determination of MIC, disc diffusion assay, microdilution assay, and spot assay. The MIC values obtained against fluconazole (azole), amphotericin B (polyene), and caspofungin (echinocandin) for these strains were similar to those for the wild type. These results suggested that strains carrying truncated Chr4 remain sensitive to these drugs. Furthermore, microdilution and disc diffusion assays were carried out for all eight strains to assess their responses to antifungals. The disc diffusion assay results showed slight variation in the responses to antifungals. Most of the generated strains showed slightly less sensitivity to the drugs compared to the wild type (Table 4). Interestingly, when strains were spotted on the plates containing increasingly higher concentrations of fluconazole, the *Candida* strain carrying truncation 4 (1201.989 kbp position on Chr4) showed better growth at fluconazole 8–64 μg/mL. However, the MIC value of this strain was the same as that of wild type (0.19 μg/mL for fluconazole). These findings indicated that the MIC values cannot predict the tolerance of a specific strain to antifungals. Previous observations revealed that antifungal tolerance operates at different pathways in this fungal pathogen. For example, the Rim pathway participates in antifungal tolerance through Hsp90p and Ipt1p [43]. However, tolerance to caspofungin can be mediated through the regulation of *FSK* gene expression and cell wall remodeling [44]. The antifungal tolerance could also be a subpopulation effect in which clinical isolates of *C. albicans* grow beyond the MIC. This extended growth is often associated with persistent candidemia [45]. Fluconazole is commonly used as the first-line drug for the treatment and management of *Candida* infections. Therefore, fluconazole tolerance can adversely affect the treatment of *C. albicans* bloodstream infections, and the patients could be at a high risk of morbidity and mortality [46].

Chr4 does not contain any drug resistance gene except the ORF *Orf19.3120* (coordinates: 1538056–1539795 on Chr4), which encodes a half-size PDR-subfamily ABC transporter. To assess the role of *Orf19.3120* in drug resistance, truncations 7 and 8 were performed on either side of this ORF at 1529.969 kbp and 1542.907 kb positions, respectively. The strain carrying truncation 8 grew slightly better than the strain carrying truncation 7 (Fig. 7). The overexpression of specific drug transporter genes, such as *CDR1* and *CDR2* produced a drug-resistant phenotype [47]. Hence, we overexpressed *Orf19.3120* in *Candida* strain CAF4-2 and assessed its phenotype on the plate containing fluconazole.

Interestingly, the *Candida* strain with overexpressed *Orf19.3120* showed an optimal growth in the presence of fluconazole (Fig. 8), thereby suggesting that *Orf19.3120* may have a minor role in drug resistance. Conversely, the strain lacking this ORF was sensitive to fluconazole in the presence of silver nanoparticles [48].

Genomic plasticity is one of the major characteristics of *C. albicans* [49]. Rapid unusual genome changes in this pathogen might occur when mitotic cells are propagated in vitro as well as in vivo. Also, aneuploidy is detected in some pathogenic fungi. These phenomena suggested that variations in chromosome organization and copy number are common, rapid, and efficient means to generate diversity in response to stressful conditions, including the presence of drugs [49]. Moreover, Chr4 trisomy has been reported in a clinical isolate with a putative role in elevated fluconazole resistance. However, Chr4 trisomy failed to increase fluconazole resistance in the background of standard *Candida* strain SC5314 [50] used for sequencing the *Candida* genome. Furthermore, some clinical isolates harbor trisomy of Chr4 and Chr7 but their roles in drug resistance are not yet ascertained [51]. In addition, the presence of Chr4 monosomy in any clinical isolates of *C. albicans* and its association with drug resistance also has not been reported. Therefore, Chr4 monosomy or trisomy cannot be considered responsible for drug resistance. However, in specific genetic backgrounds with predisposing mutations or genomic changes, these conditions could be implicated in drug resistance.

## Conclusion

In summary, we performed systematic chromosomal truncations of Chr4 of *Candida albicans* and assessed their responses to antifungals. The partial or segmental aneuploidies generated were challenged against three classes of antifungals, such as azoles, polyenes, and echinocandin. All the strains carrying truncated Chr4 were sensitive to these antifungals, similar to that for the wild type strain. However, some truncations exhibited a highly tolerant phenotype against fluconazole, a front-line antifungal drug. Drug tolerance was also observed when the strains were incubated in fluconazole for > 2 days. Therefore, *C. albicans* uses Chr4 as a drug-tolerant arsenal, which would benefit its propagation and colonization at different niches, posing a threat to candidiosis treatment and management. Finally, we concluded that it is unlikely that Chr4 is involved in drug resistance in this fungal pathogen. However, it may participate in developing resistance to antifungals in specific genetic backgrounds.

Nevertheless, truncations 4 and 8 would be explored in future studies to unveil the genes responsible for fluconazole tolerance. The strains will also be tested against other commonly used antifungals to deduce their efficacy with respect to these two truncations. In addition, we aim for truncations in the left portion of Chr4 to identify the chromosomal segments involved in drug resistance/tolerance. Further studies would be required to compare the genes’ expression in the strains carrying truncated Chr4, which would be valuable in understanding the *Candida* biology.

## Supplementary Information

**Additional file 1: Supplementary Table S1.** Minimum inhibitory concentration (MIC) for the strains carrying truncated chromosome 4. **Supplementary Fig. S1.** (A) Schematic diagram of plasmid pKA05 [25]. This plasmid was used as a backbone plasmid for generating truncation constructs. Mapping sequences (MS) from truncation sites of chromosome 4 were inserted at K-Xh (KpnI-XhoI) and the resulting plasmids were digested with KpnI-SacI and transformed into Candida. (B) Schematic diagram of PCR verification for chromosomal truncation. Primers P1 and P2 were designed from upstream of MS of chromosome 4 and URA3 flipper, respectively. The primers amplify PCR product of expected size only when truncation occurs at the intended site on Chr4. PBT, plasmid-borne Candida telomere. **Supplementary Fig. S2.** Disc diffusion assay for strains carrying truncated homologue of Chromosome 4. WT, wild type strain CAF4-2; Trn1 to Trn8, strains carrying truncations at 969.905 kb, 1002.852 kb; 1102.087 kb; 1201.989 kb; 1301.783 kb; 1369.883 kb; 1529.969 kb; 1542.907 kb positions on Chr4. Cells were poured along with molten 0.7% agar and disc containing (A) fluconazole (25 μg); (B) clotrimazole (10 μg); (C) itraconazole (10 μg); (D) ketokonazole (10 μg); (E) miconazole (30 μg); (F) amphotericin B (50 μg); (G) nystatin (50 μg) were placed on the plate (Himedia, Mumbai, India). Photographs were taken after 24 hr.

## Data Availability

The authors declare that all data are included in the article.

## Abbreviations

ABC: ATP-binding cassette
CGD: Candida Genome Database
CHEF: Contour-clamped homogenous electric field
Chr4: Chromosome 4
CLSI: Clinical and Laboratory Standards Institute
DMSO: Dimethyl sulfoxide
EDTA: Ethylenediaminetetraacetic acid
MFS: Major facilitator superfamily
MIC: Minimum inhibitory concentration
PCR: Polymerase chain reaction
PDR: Pleiotropic drug resistance
SD: Synthetic dextrose
WT: Wild type
YPD: Yeast extract/peptone/dextrose

## References

[1] Sudbery PE (2011). Growth of *Candida albicans* hyphae. Nat Rev Microbiol.

[2] Su C, Yu J, Lu Y (2018). Hyphal development in *Candida albicans* from different cell states. Curr Genet.

[3] Kabir MA, Hussain MA, Ahmad Z (2012). *Candida albicans*: A model organism for studying fungal pathogens. ISRN Microbiol.

[4] Mayer FL, Wilson D, Hube B (2013). *Candida albicans* pathogenicity mechanisms. Virulence.

[5] Castanheira M, Deshpande LM, Davis AP, Rhomberg PR, Pfaller MA (2017). Monitoring antifungal resistance of CLSI epidemiological values and whole-genome sequencing analysis for detection of azole resistance. Antimicrob Agents Chemother.

[6] Pappas PG, Lionakis MS, Arendrup MC, Ostrosky-Zeichner L, Kulberg BJ (2018). Invasive candidiasis. Nat Rev Dis Primers.

[7] Cowen LE, Sanglard D, Howard SJ, Rogers PD, Perlin DS (2014). Mechanisms of antifungal drug resistance. Cold Spring Harb Perspect Med.

[8] Morschhäuser J (2016). The development of fluconazole resistance in *Candida albicans* - an example of microevolution of a fungal pathogen. J Microbiol.

[9] Grela E, Zdybicka-Barabas A, Pawlikowska-Pawlega B, Cytrynska M, Wlodarczyk M, Grudzinski W, Luchowski R, Gruszecki WI (2019). Modes of the antibiotic activity of amphotericin B against *Candida albicans*. Sci Rep.

[10] Whaley SG, Berkow EL, Rybak JM, Nishimoto AT, Barker KS, Rogers PD (2017). Azole antifungal resistance in *Candida albicans* and emerging non-albicans *Candida* species. Front Microbiol.

[11] Pristov KE, Ghannoum MA (2019). Resistance of *Candida* to azoles and echinocandins worldwide. Clin Microbiol Infect.

[12] Zarnowski R, Sanchez H, Covelli AS, Dominguez E, Jaromin A, Bernhardt J, Mitchell KF, Heiss C, Azadi P, Mitchell A, Andes DR (2018). *Candida albicans* biofilm-induced vesicles confer drug resistance through matrix biogenesis. PLoS Biol.

[13] Prasad R, Nair R, Banerjee A (2019). Emerging mechanism of drug resistance in *Candida albicans*. Prog Mol Subcell Biol.

[14] Redhu AK, Shah AH, Prasad R (2016). MFS transporters of Candida species and their role in clinical drug resistance. FEMS Yeast Res.

[15] Flowers SA, Colón B, Whaley SG, Schuler MA, Rogers PD (2015). Contribution of clinically derived mutations in *ERG11* to azole resistance in *Candida albicans*. Antimicrob Agents Chemother.

[16] Morio F, Pagniez F, Lacroix C, Miegeville M, Pape PL (2012). Amino acid substitutions in the *Candida albicans* sterol Δ^5,6^-desaturase (Erg3p) confer azole resistance: characterization of two novel mutants with impaired virulence. J Antimicrob Chemother.

[17] Hope WW, Tabernero L, Denning DW, Anderson MJ (2004). Molecular mechanisms of primary resistance to flucytosine in *Candida albicans*. Antimicrob Agents Chemother.

[18] Spettel K, Barousch W, Makristathis A (2019). Analysis of antifungal resistance genes in *Candida albicans* and *Candida glabrata* using next generation sequencing. PLoS One.

[19] Ramírez-Zavala B, Manz H, Englert F, Rogers PD, Morschhäuser J (2018). A hyperactive form of the zinc cluster transcription factor Stb5 causes *YOR1* overexpression and beauvericin resistance in *Candida albicans*. Antimicrob Agents Chemother.

[20] Nishimoto AT, Zhang Q, Hazlett B, Morschhäuser J, Rogers PD (2019). Contribution of clinically derived mutations in the gene encoding the zinc cluster transcription factor Mrr2 to fluconazole antifungal resistance and *CDR1* expression in *Candida albicans*. Antimicrob Agents Chemother.

[21] Rustchenko EP, Howard DH, Sherman F (1994). Chromosomal alterations of *Candida albicans* are associated with the gain and loss of assimilating functions. J Bacteriol.

[22] Janbon G, Sherman F, Rustchenko E (1998). Monosomy of a specific chromosome determines L-sorbose utilization: a novel regulatory mechanism in *Candida albicans*. Proc Natl Acad Sci USA.

[23] Perepnikhatka V, Fischer FJ, Niimi M, Baker RA, Cannon RD, Wang YK, Sherman F, Rustchenko E (1999). Specific chromosome alterations in fluconazole-resistant mutants of *Candida albicans*. J Bacteriol.

[24] Kabir MA, Ahmad A, Greenberg J, Wang YK, Rustchenko E (2005). Loss and gain of chromosome 5 controls growth of *Candida albicans* on sorbose due to dispersed redundant negative regulators. Proc Natl Acad Sci USA.

[25] Reddy PK, Pullepu D, Dhabalia D, Udaya Prakash SM, Kabir MA (2020). *CSU57* encodes a novel repressor of sorbose utilization in opportunistic human fungal pathogen *Candida albican*. Yeast.

[26] Fonzi WA, Irwin MY (1993). Isogenic strain construction and gene mapping in *Candida albicans*. Genetics.

[27] Gough JA, Murray NE (1983). Sequence diversity among related genes for recognition of specific targets in DNA molecules. J Mol Biol.

[28] Sherman F, Guthrie C, Fink GR (2002). Getting started with the yeast. Methods in enzymology. Guide to yeast genetics and molecular biology.

[29] Sambrook J, Russell DW (2001). Molecular cloning: A laboratory manual*.* Cold Spring Harbor Laboratory Press.

[30] Birnboim HC, Doly J (1979). A rapid alkaline extraction procedure for screening reombinant plasmid DNA. Nucleic Acids Res.

[31] Hinnen A, Hicks JB, Fink GR (1978). Transformation of yeast. Proc Natl Acad Sci USA.

[32] Kabir MA, Rustchenko E (2005). Determination of gaps by contig alignment with telomere-mediated chromosomal fragmentation in *Candida albicans*. Gene.

[33] Morschhäuser J, Michel S, Staib P (1999). Sequential gene disruption in *Candida albicans* by FLP-mediated site-specific recombination. Mol Microbiol.

[34] McEachern MJ, Hicks JB (1993). Unusually large telomeric repeats in the yeast *Candida albicans*. Mol Cell Biol.

[35] Cannon RD, Jenkinson HF, Shepherd MG (1992). Cloning and expression of *Candida albicans ADE2* and proteinase genes on a replicative plasmid in *C. albicans* and *Saccharomyces cerevisiae*. Mol Gen Genet.

[36] Skrzypek MS, Binkley J, Binkley G, Miyasato SR, Simison M, Sherlock G (2017). The Candida Genome Database (CGD): incorporation of Assembly 22, systematic identifiers and visualization of high throughput sequencing data. Nucleic Acids Res.

[37] Clinical and Laboratory Standards Institute (2018). Performance standards for antifungal susceptibility testing of yeasts.

[38] Ostrosky-Zeichner L, Rex JH, Pfaller MA, Diekema DJ, Alexander BD, Andes D, Brown SD, Chaturvedi V, Ghannoum MA, Knapp CC, Sheehan DJ, Walsh TJ (2008). Rationale for reading fluconazole MICs at 24 hours rather than 48 hours when testing *Candida* spp. by the CLSI M27-A2 standard method. Antimicrob Agents Chemother.

[39] Clinical and Laboratory Standards Institute (2018). Method for antifungal disk diffusion susceptibility testing of yeasts.

[40] Gaur M, Choudhury D, Prasad R (2005). Complete inventory of ABC proteins in human pathogenic yeast, *Candida albicans*. J Mol Microbiol Biotechnol.

[41] Berman J (2016). Ploidy plasticity: A rapid and reversible strategy for adaptation to stress. FEMS Yeast Res.

[42] Abbey D, Hickman M, Gresham D, Berman J (2011). High-resolution SNP/CGH microarrays reveal the accumulation of loss of heterozygosity in commonly used *Candida albicans* strains. G3.

[43] Garnaud C, García-Oliver E, Wang Y, Maubon D, Bailly S, Despinasse Q, Champleboux M, Govin J, Cornet M (2018). The Rim pathway mediates antifungal tolerance in *Candida albicans* through newly identified Rim101 transcriptional targets, including Hsp90 and Ipt1. Antimicrob Agents Chemother.

[44] Yang F, Zhang L, Wakabayashi H, Myers J, Jiang Y, Cao Y, Jimenez-Ortigosa C, Perlin DS, Rustchenko E (2017). Tolerance to caspofungin in *Candida albicans* is associated with at least three distinctive mechanisms that govern expression of *FKS* genes and cell wall remodeling. Antimicrob Agents Chemother.

[45] Rosenberg A, Ene IV, Bibi M, Zakin S, Segal ES, Ziv N, Dahan AM, Colombo AL, Bennett RJ, Berman J (2018). Antifungal tolerance is a subpopulation effect distinct from resistance and is associated with persistent candidemia. Nat Commun.

[46] Levinson T, Dahan A, Novikov A, Paran Y, Berman J, Ben-Ami R (2021). Impact of tolerance to fluconazole on treatment response in *Candida albicans* bloodstream infection. Mycoses.

[47] Prasad R, Balzi E, Banerjee A, Khandelwal NK (2019). All about CDR transporters: Past, present, and future. Yeast.

[48] Dhabalia D, Ukkund SJ, Syed ST, Uddin W, Kabir MA (2020). Antifungal activity of biosynthesized silver nanoparticles from *Candida albicans* on the strain lacking the *CNP41* gene. Mater Res Express.

[49] Selmecki A, Forche A, Berman J (2010). Genomic plasticity of the human fungal pathogen *Candida albicans*. Eukaryot Cell.

[50] Anderson MZ, Saha A, Haseeb A, Bennett RJ (2017). A chromosome 4 trisomy contributes to increased fluconazole resistance in a clinical isolate of *Candida albicans*. Microbiology (Reading).

[51] Hirakawa MP, Martinez DA, Sakthikumar S, Anderson MZ, Berlin A, Gujja S, Zeng Q, Zisson E, Wang JM, Greenberg JM, Berman J, Bennett RJ, Cuomo CA (2015). Genetic and phenotypic intra-species variation in *Candida albicans*. Genome Res.

