# Supplementary material for: Systematic truncations of chromosome 4 and their responses to antifungals in Candida albicans
Source: J Genet Eng Biotechnol. 2021 Jun 21;19:92. doi: 10.1186/s43141-021-00197-0 (PMC8217416; doi:10.1186/s43141-021-00197-0)
Supplement: Supplementary file 1 — Additional file 1: Supplementary Table S1. Minimum inhibitory concentration (MIC) for the strains carrying truncated chromosome 4. Supplementary Fig. S1. (A) Schematic diagram of plasmid pKA05 [25]. This plasmid was used as a backbone plasmid for generating truncation constructs. Mapping sequences (MS) from truncation sites of chromosome 4 were inserted at K-Xh (KpnI-XhoI) and the resulting plasmids were digested with KpnI-SacI and transformed into Candida. (B) Schematic diagram of PCR verification for chromosomal truncation. Primers P1 and P2 were designed from upstream of MS of chromosome 4 and URA3 flipper, respectively. The primers amplify PCR product of expected size only when truncation occurs at the intended site on Chr4. PBT, plasmid-borne Candida telomere. Supplementary Fig. S2. Disc diffusion assay for strains carrying truncated homologue of Chromosome 4. WT, wild type strain CAF4-2; Trn1 to Trn8, strains carrying truncations at 969.905 kb, 1002.852 kb; 1102.087 kb; 1201.989 kb; 1301.783 kb; 1369.883 kb; 1529.969 kb; 1542.907 kb positions on Chr4. Cells were poured along with molten 0.7% agar and disc containing (A) fluconazole (25 μg); (B) clotrimazole (10 μg); (C) itraconazole (10 μg); (D) ketokonazole (10 μg); (E) miconazole (30 μg); (F) amphotericin B (50 μg); (G) nystatin (50 μg) were placed on the plate (Himedia, Mumbai, India). Photographs were taken after 24 hr. [file 43141_2021_197_MOESM1_ESM.docx]

**Supplementary Materials**

**Supplementary Table S1.** Minimum inhibitory concentration (MIC) for the strains carrying truncated chromosome 4.

| Truncation | Truncation site | FLC (µg/ml) | AP (µg/ml) | CAS (µg/ml) |
| --- | --- | --- | --- | --- |
| Wild type | No truncation | 0.19 | 0.75 | 0.064 |
| Trn.1 | 969.925 | 0.25 | 1.0 | 0.125 |
| Trn.2 | 1002.852 | 0.38 | 0.75 | 0.064 |
| Trn.3 | 1102.087 | 0.38 | 0.75 | 0.19 |
| Trn.4 | 1201.989 | 0.19 | 0.75 | 0.19 |
| Trn.5 | 1301.783 | 0.25 | 0.75 | 0.19 |
| Trn.6 | 1369.883 | 0.19 | 0.75 | 0.25 |
| Trn.7 | 1529.969 | 0.25 | 0.75 | 0.094 |
| Trn.8 | 1542.907 | 0.19 | 1.0 | 0.19 |

FLC, fluconazole; AP, amphotericin B; CAS, caspofungin.


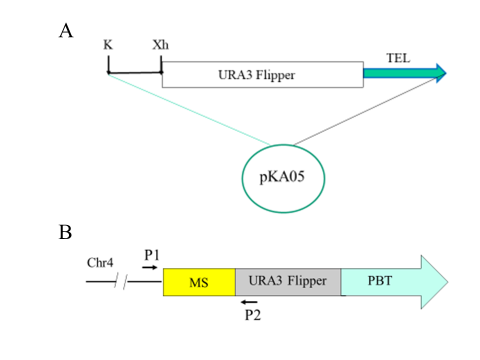


**Supplementary Fig. S1** **(A)** Schematic diagram of plasmid pKA05 (Ref. 26). This plasmid was used as a backbone plasmid for generating truncation constructs. Mapping sequences (MS) from truncation sites of chromosome 4 were inserted at K-Xh (*Kpn*I-*Xho*I) and the resulting plasmids were digested with *Kpn*I-*Sac*I and transformed into *Candida*. (**B)** Schematic diagram of PCR verification for chromosomal truncation. Primers P1 and P2 were designed from upstream of MS of chromosome 4 and *URA3* flipper, respectively. The primers amplify PCR product of expected size only when truncation occurs at the intended site on Chr4. PBT, plasmid-borne *Candida* telomere.

A


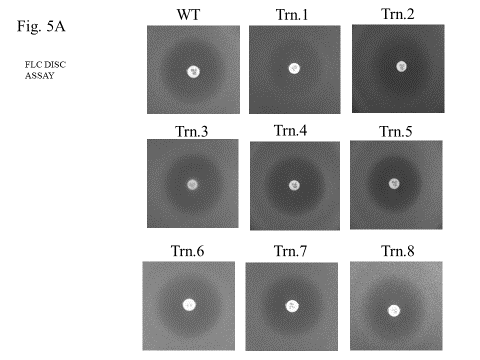


B


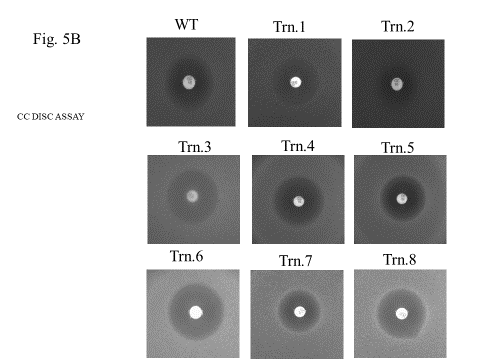


C


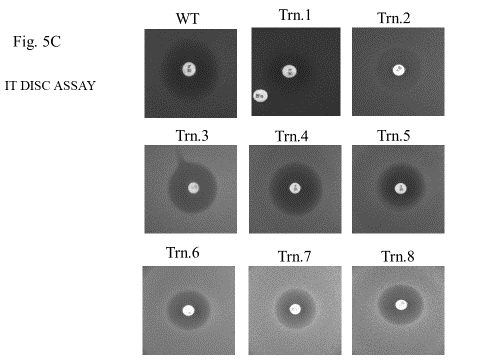


D


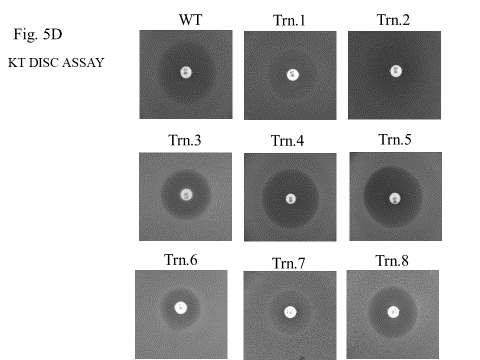


E


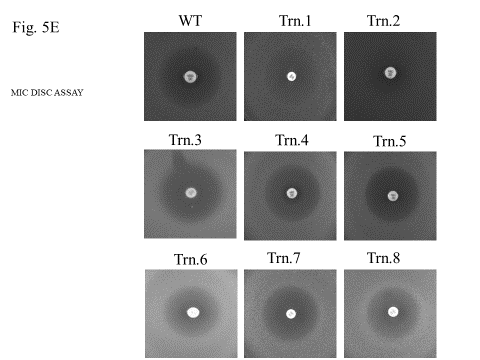


F


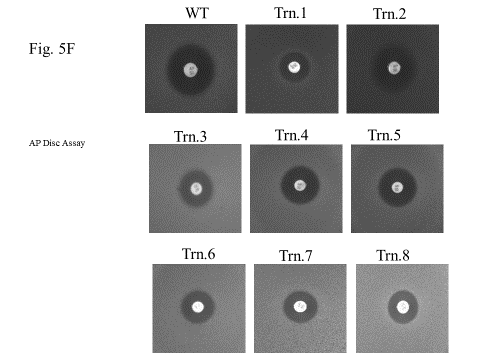


G


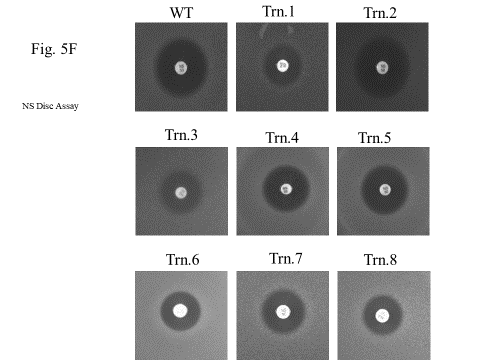


**Supplementary Fig. S2** Disc diffusion assay for strains carrying truncated homologue of Chromosome 4. WT, wild type strain CAF4-2; Trn1 to Trn8, strains carrying truncations at 969.905 kb, 1002.852 kb; 1102.087 kb; 1201.989 kb; 1301.783 kb; 1369.883 kb; 1529.969 kb; 1542.907 kb positions on Chr4. Cells were poured along with molten 0.7% agar and disc containing (A) fluconazole (25 µg); (B) clotrimazole (10 µg); (C) itraconazole (10 µg); (D) ketokonazole (10 µg); (E) miconazole (30 µg); (F) amphotericin B (50 µg); (G) nystatin (50 µg) were placed on the plate (Himedia, Mumbai, India). Photographs were taken after 24 hr.
